# Child perception and parent’s perception about child sleep quality

**DOI:** 10.5935/1984-0063.20200107

**Published:** 2021

**Authors:** Ana Maria Gomes, Mariana Costa Martins

**Affiliations:** Universidade Autónoma de Lisboa, Departamento de Psicologia e Centro de Investigação em Psicologia - Lisboa - Portugal.

**Keywords:** Sleep assessment, Children, Sleep Quality, Sleep Disorder Index

## Abstract

**Introduction:**

Sleep is a physiological necessity that interferes with the activity during the day. This study aimed to analyze child perception about sleep quality and compare it with parent’s perception about the quality of their children’s sleep, and to investigate the sleep quality of Portuguese schoolchildren. Analyze the differences between the sexes and the type of school attended.

**Material and Methods:**

Cross-sectional study, quantitative methodology. The results of two questionnaires, the Pittsburgh sleep quality index (PSQI) answered directly by the children, and the children’s sleep habits questionnaire (CSHQ), answered by the parents of 883 children, were analyzed and compared.

**Results:**

PSQI reveals good sleep quality, which contradicts the results of CSHQ. The CSHQ indicates a mean sleep deterioration index (IPS) value of 46.12 (above the cutoff point, 44) indicating that on average the children in this sample have poor sleep quality. There is no significant difference between girls and boys regarding IPS. There is a significant difference in the level of daytime drowsiness (p=.018), girls wake up moodier (p=.011), have more difficulty getting out of bed in the morning (p=.019), and take longer to fully awaken than boys (p=.004).

**Conclusion:**

The data show that children seem to have poor sleep quality and that they erroneously evaluate it, but these same data should be read with caution since the reason for the different perception between parents and children is not known.

## INTRODUCTION

Sleeping is a homeostatic necessity, fundamental for the health of the human being for an essential and adequate organic and psychological functioning^27, 30^. Lack of sleep has negatives repercussions on a variety of aspects, including stress^4^, de pression^4, 14^, self-regulation, self-esteem^14, 30^, academic performance^15, 17, 30^, and neurocognitive functioning (e.g., memory)^26^. This impact is very visible in children, particularly children of middle school age^18, 24^.

These domains (affected by sleep problems) become more relevant when taking in consideration children who are more vulnerable than most to these problems (i.e., sleep deprivation). Including those who have suffered from childhood trauma, and/ or suffer from neurological pathologies or cognitive problems^28^. Another factor that may contribute to poor sleep in childhood is the social and ecological environment. Hence, low social-economic conditions are a risk factor in terms of sleep quality^10^.

Sleep is a phenomenon that is not immune or independent of cultural and social factors. Some cultures value a good night of sleep more than others. Sleep time being required, it is sometimes devalued in certain cultural and social contexts. Research in Europe and the United States shows that most parents evaluate the importance of sleep very positively, yet 90% of children sleep less than recommended^8^. We live in a sleep-deprived society, among both adults and children and where sleep disturbances begin to emerge as quite expressive. In fact, sleep restriction is increasingly common in industrialized societies because of the extension of work throughout the 24 hours of the day. Some recent studies assume that children’s sleep duration has decreased in the last few decades by about 30 to 60 minutes, and it is crucial to understand the characteristics and restrictive factors for sleep during childhood, while integrating in this understanding the dynamics of parental support behaviors^20^.

Hence, this study is based on the concept(s) of quality/ quantity of sleep, and focus on the importance of these domains. Sleep quality and sleep duration are two different domains of sleep, although both overlap and differentiate. Sleep quality refers to the subjective indexes of how sleep is experienced, includes the feeling of satisfaction and being rested upon awakening. Intrinsic to this sense of rest and satisfaction is also the concept of sleep hygiene, which portraits a variety of different practices and habits that are necessary to have good quality of nocturnal sleep and allow for daytime alertness. The amount of sleep is a bigger influence on the possibility of daytime sleepiness, changes in emotional state, behavioral and cognitive functions^13^.

Adequate sleep duration for healthy individuals who do not suffer from any sleep disturbance varies greatly at the level of the developmental cycle. For children of preschool age, the recommended hours of sleep range between 10 and 13 hours and for children of school age it is between 9 and 11 hours. Thus, sufficient sleep duration requirements vary throughout the life span and from person to person^16^. Individuals who habitually sleep out of the normal pattern may be showing signs or symptoms of health problems and this may compromise their well-being^2^. Sleep problems during childhood and adolescence are frequent and sometimes they are not transient. Some studies report that sleep problems do not disappear or diminish at any rate with ageing, and they may become chronic^6^. It is of great concern that many parents still underestimate their children’s sleep problems and the seriousness of their consequences^5, 11^.

Palmstierna et al. (2008)^22^ present a study in which children have better quality sleep as they age, with individual sleep patterns becoming more stable. The child’s perception of poor-quality sleep is influenced by the child’s temperament and the mother’s foreign origin. It is important to ascertain the quality of sleep, especially for children, so it is important to know how to evaluate it. In this way, it is noted that the assessment made, specifically the informers (for example, the parents or the child) to whom it will be reported, could determine a more positive or negative assessment of the quality of infant sleep and decisively the intervention to be adopted.

Despite the proven importance of sleep as an area of study for the overall health and well-being of children, there is a gap in the literature regarding the analysis and detailed comparison of different instruments and different sources of information regarding sleep quality^22^. One of the objectives of this study is precisely to start addressing this gap by comparing the child’s and parent’s perception of sleep. Since the literature already points out that different sources of information may reveal a trend towards different assessments of sleep quality, it will be important to study and test the hypothesis of this investigation: if children, due to their natural vulnerability to a still developing emotional and social communication and sense of self, are not as perceptive and sensitive in identifying possible disturbances regarding the quality of their own sleep, in comparison with adults who know them really well, as their parents. For this reason, children are expected to reveal less levels of sleep disturbances, in comparison with their parents.

Parents’ perception of their children’s quality of sleep is dominated by whether or not they wake up at night. Therefore, if there are frequent night awakenings, it is natural for parents to classify their children’s sleep quality as inferior. However, if parents already consider their child’s quality of sleep to be low, this may make them aware of their child’s awakening and lead them to report it as more frequent^22^. There thus seems to be a strong association between night awakening and the quality of sleep from the point of view of parental evaluation, for it is the frequency of night awakening that is the main factor by which parents judge the quality of their children’s sleep.

Sleep problems in children are often diagnosed based on parents’ reactions. It is the parents’ perception of the quality of their children’s sleep that begins the analysis of their children’s sleep quality. When the parents’ sleep is affected by their children’s awakenings, the identification of the child’s sleep difficulties reported by the parents arises^23^.

There is a reciprocal influence between the quality of sleep of children and the quality of sleep of parents. When children have a poor quality of sleep, it implies that the parents have it too. Parents with a poor quality of sleep reveal greater cognitive or somatic arousal before sleeping when their children have sleep difficulties. Poor quality sleep by parents can be associated with chronic insomnia problems, and their mood is also mediated by their children’s quality of sleep^29^. Authors such as Mazza et al. (2020)^19^ analyzed and compared children’s self-perception about their sleep and estimates of the quality of sleep made by their parents, concluded that there are discrepancies between the two denoting insufficient agreement on sleep duration and night wakefulness^19^.

In this line of thought, it will be interesting to see if the way children evaluate their sleep differs from the parents’ assessment of their own children’s sleep. It is important to take into account that parents and children differ in how they evaluate their behavior and emotions, and meta-analysis and data previously gathered by other authors point out that when concerned with externalization problems (more observable) compared to internalization (less observable) there is greater agreement (i.e., less discrepancy) and the latter is larger for the 6 to 11 age group than for adolescents. These authors therefore gathered a considerable database, noting that the correlation between the parents’ report and the child/ adolescent’s own report did not go beyond a low association, with a correlation coefficient of 0.22^9^.

In addition to this frst objective, a descriptive analysis of the sleep quality of the population associated to our sample was performed (in this case, the sleep quality Portuguese children was assessed). In order to have a more in depth understanding of the levels of sleep disturbance suffered by our children. It is sought as well to be investigated the differences in the quality of sleep related to gender and to the type of school (public or private).

## MATERIAL AND METHODS

### Participants

A non-probabilistic and representative sampling was used, in order to study the targeted population of this investigation. The sample consisted of children aged between 6 and 10 years old, attending the 1^st^ cycle of basic education, constituting a total of 883 children. The sample is quite representative because it includes six schools, in which three are public and three are private. All of which are located in the center of Portugal, Lisbon, and Leiria. The inclusion criteria are to attend a public or private school in the 1^st^ cycle of basic education and be between 6 and 10 years old. Exclusion factors are being over 10 years old or having some severe cognitive impairment that prevents from understanding and responding to the instruments.

Participants are divided into two major groups, one of which is the group of children who responded to the Pittsburgh sleep quality index (PSQI) (n=1,109). The same parents of these children who answered the children’s sleep habits questionnaire (CSHQ-PT) totaling 883 responses, and a mortality of 226 subjects (20.38%), since not all parents of the initial 1,109 children evaluated, returned the questionnaires and/or signed the informed consent. These 883 children assessed by their parents regarding sleep quality were divided into 437 (49.5%) boys and 446 girls (50.5%) from public (n=403) and private (n=480) schools. The mean age of participants (children) was 8 years±1.241 with 99.5%, ranging from 6 to 10 years of age.

### Instruments

#### Pittsburgh sleep quality index (PSQI)

The PSQI was then used to study the quality of sleep evaluated by children, which was developed^9^ and adapted in the Portuguese version. Its translation into Portuguese was performed^7^. It is considered stable to evaluate sleep quality, since it is accessible and identifies whether the subjects studied sleep well or poorly. Structurally it consists of 19 questions, with 15 multiple choice items that refer to the frequency of sleep disturbances and subjective quality of sleep, and 4 items that refer to bedtime and wake time and sleep duration and latency. It is considered an instrument of quick administration, easy application, reliable and suitable for clinical research in different age groups (between 6 and 90 years of age) including clinical and non-clinical population^12^. This instrument in general allows assessing the sleep quality in adults and children.

#### Children’s sleep habits questionnaire (CSHQ-PT)

The instrument used to study the perception that parents have about the sleep quality of their children was the CSHQ-PT, adapted and validated it for the Portuguese population, having made a broad characterization of sleep habits in children from 2 to 10 years of age. The internal consistency of CSHQ-PT (Cronbach’s α) was 0.78 for the total scale and ranged from 0.44 to 0.74 for the subscales. The test-retest reliability for the subscales (Pearson correlations, n=58) ranged from 0.59 to 0.85. The CSHQ-PT showed psychometric properties that are comparable to other countries’ versions and suitable for the screening of sleep disorders in children^25^.

### Procedures and statistical analysis

The sample collection was carried out by a team of three researchers, who contacted all the school principals and, after meetings, obtained the authorization to proceed with the investigation. This collection took place between the months of March and June 2016.

Data was collected in the classroom with the assistance of the teacher. All participants took home an informed consent that was signed by the parent or legal guardian of the child. In this informed consent, the objectives of the study and the procedures were explained, stating that no images will be collected and all statistical data will be worked on without identifying the students’ names, as each student will be given a code, not allowing at any time that these identified. With absolute anonymity and confidentiality of the data of each of the participants. Authorization was requested to collect and archive the data without the use of names to maintain the students’ absolute anonymity. It was said that all data collected will be used exclusively for the purpose of scientific research and will respect the data protection rules of scientific ethics and European general law, in accordance with the terms of Law 67/98, of 26 October and EU Regulation 2016/679 of the European Parliament and of the Council.

Descriptive statistics were performed, with mean, standard deviation, asymmetry, and kurtosis of the 7 components and overall PSQI index in the global sample. For the comparison between the two groups, the non-parametric Mann-Whitney U test was performed. In order to carry out the analysis of the children’s sleep habits questionnaire (CSHQ-PT), descriptive statistics were also performed, with mean, standard deviation, asymmetry, and kurtosis of the 33 CSHQ-PT items in the global sample. For comparison between the two groups, the non-parametric Mann-Whitney U test was used.

## RESULTS

As shown in Table 1, PSQI had mean value of the overall score is 4.05, which is significantly below the cutoff of 5, t(859)=- 9.92, *p*<.001, while at the same time (37.1%) scored equal to or greater than 5. Analyzing in depth gender differences, there was no (statistically) significant difference for the Global PSQI (U=90912, Z=-.422, *p*=.673). The boys registered higher scores than the girls in sleep latency and sleep duration, with statistically significant differences (U=83227.50, Z=-2.727, *p*=.006 and U=90464, Z=-2.564, *p*<.010, respectively), which did not happen for the remaining PSQI’s dimensions.

**Table 1:** PSQI data.

		Global PSQI	Subjective Sleep Quality	Sleep Latency	Sleep duration	Sleep efficiency	Sleep Disorders	Use of sleep medication	Daytime disfunction
Mean (M)	4.05	.63	.89	.02	.06	1.29	.63	.56	
Standard deviation (SD)	2.803	.794	1.018	.203	.309	.597	.794	.904	
Percentiles	25	2	0	0	0	0	1	0	0
	50	4	0	0	0	0	1	0	0
	75	6	1	2	0	0	2	1	1
Girls	M	3.96	.62	.79	.00	.04	1.30	.62	.60
	SD	2.664	.775	.944	.068	.199	.612	.775	.919
Boys	M	4.14	.63	1.00	.04	.08	1,29	.63	.51
	SD	2.939	.814	1.079	.280	.390	.582	.814	.893
U Mann-Whitney -G		90912.00	92389.50	83227.50**	90464***	91832.50	92386.50	92389.50	86769.50
Public School	M	4.23	.68	.91	.01	.04	1.30	.68	.62
	SD	2.837	.870	1.025	.154	.232	.624	.870	.938
Private School	M	3.91	.58	.88	.03	.07	1.29	.58	.51
	SD	2.730	.726	1.014	.235	.358	.575	.726	.874
U Mann-Whitney (S)	85322	88206	89410	90023	89533	90168	88206	85418.50	

When comparing the results of children in public and private schools, no statistically significant difference was found for the Global PSQI (U=85322, Z=-1.638, p*˂*.101), nor for the respective domains of this measure.

As shown in Table 2, the mean value of the sleep disorder index (IPS, obtained from CSHQ-PT) is 46.12, which is significantly above the cutoff point of 44.00, t(882)=8.11, *p˂*.001. There was no statistically significant difference between girls and boys at the IPS level (U=91137.50, Z=-1.668, *p˂*.095). Gender difference occurred for the daytime drowsiness dimension (U=88506, Z=-2.373, *p˂*-.018), specifically through the items “wakes up grumpy” (U=88447.50, Z=-2.540, *p*<.011), “difficulty in getting out of bed in the morning” (U=89192, Z=-2.350, *p˂*.019) and delay in fully awakening (U=87904, Z=- 2,910, *p˂*.004 ), with girls having higher scores. It was also observed that they wet the bed at night more often in a (statistically) significant way (U=93505, Z=-2.401, *p˂*.016).

**Table 2:** CSHQ-PT data.

	Mean	Standard deviation (SD)	G-Girls		G-Boys		U MannWhitney -G	Public School		Private School		U MannWhitney (S)
	(M)		M	SD	M	SD		M	SD	M	SD	
IPS	46.12	7.78	46.29	7.28	45.95	8.26	91137.50	46.76	8.00	45.59	7.56	87564.00*
Resistance in going to bed	8.02	2.58	8.08	2.52	7.97	2.63	93521.50	8.27	2.75	7.82	2.41	87814.00*
Start of sleep	1.95	0.88	1.96	0.87	1.93	0.88	95573.00	2.02	0.85	1.88	0.90	88199.50*
Length of sleep	3.78	1.17	3.84	1.21	3.73	1.13	92226.00	3.78	1.17	3.79	1.18	95925.50
Sleep related Anxiety	5.86	2.06	5.97	2.06	5.76	2.05	90510.00	6.04	2.10	5.71	2.01	87359.50***
Night awakenings	3.71	1.13	3.65	1.01	3.77	1.24	94833.00	3.76	1.11	3.66	1.15	89710.50*
Parasomnias	8.58	1.74	8.43	1.55	8.72	1.90	91106.50	8.76	1.82	8.42	1.65	85289.00****
Respiratory sleep disorder	3.42	0.90	3.38	0.86	3.46	0.94	94419.50	3.48	0.90	3.38	0.90	88422.00****
Daytime sleepiness	13.66	3.09	13.91	3.17	13.41	3.00	88506.00*	13.59	3.19	13.72	3.01	94122.00

Regarding the type of school, there are differences, with the public ones having higher scores in the IPS, in a statistically significant way (U=87564, Z=-2.429, *p˂*.015), and the same happens for the dimensions of resistance in going to bed (U=87814, Z=-2.460, *p˂*.014), sleep related anxiety (U=87359.50, Z=-2.560, *p˂*.010), nocturnal awakenings (U=89710.50, Z=-2,155, *p˂*.031), parasomnias (U=85289, Z=-3.125, *p˂*.002), and respiratory sleep disorders (U=88422, Z=-2.852, *p˂*.004).

## DISCUSSION

An analysis of the data coming from measures of sleep quality was proceeded. In doing so, and with regard to the subjective assessment of children about their sleep, they were found to have positively assessed it, with a value below the cutoff point for the Global PSQI. Such results however, should be interpreted while keeping in mind that a substantial percentage of children (over a third) reported having poor sleep. This leads to taking in consideration a great variability in the quality of sleep among children. There is, in fact, children who assume that they sleep well but also others who report that they sleep poorly, and the results should be interpreted with care.

The perception of parents about the quality of their children’s sleep, the mean value of CSHQ-PT is below the cutoff point, which points to poor sleep quality. It should be noted that the results of the present investigation regarding the CSHQ-PT are similar to those presented by previous authors^3^, with these studies finding mean values of 47.0 and 47.59, that is, identical to those found in the present study (46.12), which gives credibility to our data.

Following these findings, there is a discrepancy between what children perceive about the quality of their sleep, and the evaluation extracted from the parents’ report. Discrepant data had already been verified for other constructs evaluated through certain scales^1^. It has been found that the discrepancy is greater for more observable behaviors compared to less observable behaviors, leading us to the question of whether the problems related to sleep are easily accessible or not to the parents. What parents analyze about their children is a convenient source of information, but this can lead to biases compared to the child’s self-reporting. Sleep disturbances in childhood are diagnosed based on parents’ reports, and this is not very objective. If parents suffer from sleep disorders, they tend to overestimate their children’s sleep difficulties^23^. Parents who have sleeping problems report more sleep difficulties in their children than parents who sleep well. It seems that poorly sleeping nights of parents favor a negative analysis of their children’s sleep. Thus, children’s sleep interferes with their parents’ sleep and probably parents’ sleep interferes with their children’s sleep as well.

It is important to note the fact that it is not possible in our study to make a direct comparison between CSHQ and PSQI (that is, there is greater susceptibility to misinterpretation), because although both instruments evaluate the child’s sleep, they have different characteristics, for example, number of items or amplitude of the scale, so the numerical value for the cutoff point of each one is different, not to mention the fact that the two scales do not have exactly the same dimensions. Also, the results of the children (PSQI) specifically may have been conditioned by the setting in which the data were collected, since the answers may have been contaminated by colleagues or they may have responded according to something they heard.

Regarding to gender differences, girls tend to show slightly difficulty in sleep latency (PSQI) and in daytime sleepiness dimension (CSHQ-PT) despite the fact that they sleep more hours in average terms. Another relevant fact is that the CSHQ-PT reveals that children attending public schools face greater problems in sleep, which can be explained by other associated factors, such as socioeconomic level or parental or medical support.

This research contributes to a deeper understanding of children’s sleep quality and the perception that children have about their own sleep. It seems that children overestimate the quality of their sleep and parents do the opposite in relation to their children’s sleep. The quality of sleep of these children should be a concern, knowing the impact it has on health, it is urgent to develop measures to promote and improve the sleep patterns of Portuguese children. The sleep of parents should be considered in the context of the sleep of their children. Sleep interventions focused on the family or on parents’ sleep may have potential benefits for improving children’s sleep. Future studies should use more objective and reliable measures to analyze sleep quality and the relationships between this dyad.

Some limitations are identified in this study: the sample used is a non-probability sample. The PSQI as an instrument may also raise some criticism and limitations to the study, as a self-report inventory, its resulting scores can be easily exaggerated or minimized by the participant, and affected by the way the instrument is administered. Furthermore, it is a relatively new measure and consequently has not received enough investigation to determine the entirety of the psychometric measures, specially the Portuguese version. Therefore, its applicability and further validation to children can also be taken in to consideration and explored in future studies.

Taking in consideration the children in our study have a high sleep disorder index and are not aware of this fact, it would be interesting in future studies to see to what extent these children show problems in mood, behavior regulation, and their academic performance, as there are some studies that point in this direction^21^. Future studies on this subject should also consider investigating the sleep pattern of the children’s parents, to help support the literature’s hypothesis that poor sleep of the adults may interfere with their children’s sleep and vice-versa^29^.
